# Are Nepal’s water, sanitation and hygiene and menstrual hygiene policies and supporting documents inclusive of disability? A policy analysis

**DOI:** 10.1186/s12939-021-01463-w

**Published:** 2021-07-08

**Authors:** Jane Wilbur, Nathaniel Scherer, Islay Mactaggart, Govind Shrestha, Thérèse Mahon, Belen Torondel, Shaffa Hameed, Hannah Kuper

**Affiliations:** 1 London School of Hygiene & Tropical Medicine, Keppel Street, London, WC1E 7HT UK; 2 WaterAid Nepal, JM Road 10, Pabitra Tole Nakkhipot, 44700 Nepal; 3 WaterAid, 27-29 Durham Street, London, SE11 5JD UK

**Keywords:** Equiframe, Disability, Gender, Menstrual hygiene management, Policy analysis, Qualitative research

## Abstract

**Purpose:**

This study assesses the inclusion of disability in Nepal’s policy and guidance relevant to water, sanitation and hygiene (WASH), and menstrual hygiene management (MHM) in comparison to gender. We investigated both policy formulation and implementation, using the Kavrepalanchok district as a case study.

**Materials and methods:**

We used the EquiFrame framework, adapted for disability and gender, and focusing on WASH and MHM. Ten Nepali policies and guidance documents were reviewed and scored for quality against the framework, which included 21 core concepts of human rights. We also interviewed key informants to consider the inclusion of disability in the implementation of MHM interventions. We applied stratified purpose sampling to 12 government officials and service providers working in Kathmandu and the Kavrepalanchock district; conducted in-depth interviews and analysed data thematically using Nvivo 11.

**Results:**

Disability was inadequately covered within the policy documents, and MHM policy commitments for disability were almost non-existent. Participation of people with disabilities in policy development was limited; within Kavrepalanchok, policy commitments were not implemented as intended and disability service providers were unable to allocate government resources. Inadequate data on disability and MHM resulted in limited professional understanding of the issues, as service providers had no training. A narrow WASH infrastructure approach to improving MHM for people with disabilities was prioritised. MHM interventions were delivered in schools; these failed to reach children with disabilities who are often out of school. Finally, there were indications that some caregivers seek sterilisation for people with disabilities who are unable to manage menstruation independently.

**Conclusion:**

Though the Constitution of Nepal enshrines gender equality and disability inclusion, there are consistent gaps in attention to disability and MHM in policies and practice. These omit and exclude people with disabilities from MHM interventions. Investment is required to generate evidence on the MHM barriers faced by people with disabilities, which would then be drawn on to develop training on these issues for professionals to improve understanding. Subsequently, policy makers could include more concepts of human rights against disability in relevant policies and service providers could implement policy commitments as intended.

**Supplementary Information:**

The online version contains supplementary material available at 10.1186/s12939-021-01463-w.

## Introduction

In 2017, approximately 26% of women globally (1.9 billion) were of menstruating age [1]. However, many of these people – particularly those living in low- and middle-income countries (LMICs) – have poor menstrual hygiene management (MHM) (Table 1).

**Table 1:** Definition of Menstrual Hygiene Management

In 2012, the WHO and UNICEF Joint Monitoring Programme (JMP) for drinking water, sanitation, and hygiene defined MHM as: ‘Women and adolescent girls using a clean menstrual management material to absorb or collect blood that can be changed in privacy as often as necessary for the duration of the menstruation period, using soap and water for washing the body as required, and having access to facilities to dispose of used menstrual management materials. They understand the basic facts linked to the menstrual cycle and how to manage it with dignity and without discomfort or fear’ [2]. MHM also involves addressing harmful societal beliefs and taboos surrounding the issue [3].	

Inadequate MHM can negatively affect girls’ education [4] and employment [5, 6], and increase the risk of sexual violence and coercion, as well as sexual and reproductive diseases [7, 8]. A growing body of evidence highlights the additional barriers to MHM that people with disabilities face, including inaccessible water, sanitation and hygiene (WASH) facilities, lack of relevant MHM information, and inappropriate menstrual hygiene products [9, 10]. Additionally, where people with disabilities require support from others to manage their menstruation, caregivers receive limited guidance or support about how to do this [11–15]. Consequently, caregivers may feel isolated and stressed, which can result in poor MHM outcomes for the individual they support [9]. These concerns are important, as approximately 15% of the global population live with a disability [16]. However, there is a dearth of MHM interventions for people with disabilities and their caregivers, and this topic is still largely absent from global discourse on MHM. Similarly to WASH more broadly, addressing the issues around MHM requires a multi-sectoral response across health, education and disability [4, 17, 18].

The definition of MHM in Table 1, focuses on the knowledge and behaviours necessary for adequate menstrual hygiene, including personal hygiene (using clean menstrual materials, and changing and washing them when needed) and public hygiene behaviours (safe disposal of used menstrual materials). Consequently, MHM tends to be referenced within WASH policies, strategies and plans, which are vital to align national priorities with the health and wellbeing needs of the population. These documents stipulate how government, development partners, civil society and the private sector should collaborate and utilise available resources effectively and efficiently. However, in many low-and middle-income countries WASH policies are not always implemented as intended for a variety of reasons, including limited capacity of service providers to absorb allocated funds, inadequate monitoring of service provision by the government and a lack of demand from citizens [19]. Additionally, women and girls with and without disabilities commonly experience discrimination and inequalities in relation to WASH and MHM [20]. Discriminatory processes must be identified and understood and then addressed in policies and programmes.

In many parts of Nepal, menstrual behaviours are shaped by socio-cultural restrictions and taboos, which inhibit MHM [21, 22]. To combat this, Nepal has worked to integrate gender within WASH policy development and implementation, such as ensuring gender segregated toilets that include MHM facilities are constructed in schools, and ensuring women hold leadership positions in WASH management structures. However, there has not been an assessment of the extent to which the MHM needs of people with disabilities are included in the Government of Nepal’s relevant policies and supporting documents, and how they are implemented. This study aims to fill that gap.

## Methods

### Study aims and objectives

Our study sits within the wider Disabling Menstrual Hygiene Barriers research which aims to investigate and address the barriers to MHM that young people with disabilities face in the Kavrepalanchok (hereafter referred to as Kavre) district, Nepal [23]*.*

This study aimed to review the extent to which the needs of people with disabilities are included in Nepal’s policy documents related to MHM and WASH, and explore how these policy commitments are implemented, using the Kavre district as a case study. It will meet this aim by answering two linked research questions: 1) are Nepal’s policies, strategies and guidance related to MHM equally committed to disability and gender, and 2) to what extent is disability included in the implementation of MHM interventions in the Kavre district. The first question is answered by conducting a policy analysis of relevant documents, and the second by carrying out a qualitative study.

### Policy analysis: materials and methods

#### Search strategy

We followed Arskey and O’Malley’s methodological framework to review the literature: after identifying the research questions, we identified relevant studies [24]. We targeted WASH policy documents, as these incorporate personal and public hygiene behaviours (including MHM). Education and health policy documents were also included because existing literature identifies that these sectors are central to improving MHM [4, 25, 26]. Eligible documents were government policies, strategies and guidelines, and Non-Government Organisations’ (NGO) training materials to support policy implementation, which were expected to be relevant to MHM. No date range was applied, but all policies and supporting documents had to be drafted or in use.

Searches were conducted across four databases (PubMed, Medline, Global Health and Embase), but no relevant materials were identified. Consequently grey literature were identified through an online review, reference mining of existing documents analysing MHM policies and commitments in Nepal [27, 28], and through consultation with key actors involved in influencing national MHM policy and practice. Although the methodologies used in grey literature documents are not as rigorous as those in peer-reviewed articles, they are invariably developed by practitioners and service providers who have extensive knowledge of the local context, issues and possible solutions. WaterAid Nepal sourced materials that were not available online. The draft MHM policy [29] was only available in Nepali, so was translated through Google Translate. This was sent to a Nepali speaker who compared it to the original document. Any inaccuracies were corrected in the Google Translate version and this was included in the policy analysis. Table 2 presents the included materials.

**Table 2:** Included materials in the policy analysis

No.	Author	Date	Title	Document type
1	Government of Nepal, Ministry of Physical Planning and Works	2009	National Urban Water Supply and Sanitation Sector Policy	Policy
2	Ministry of health and population	2014	National Health Policy 2071, unofficial translation, draft ver.1 as of 08 Aug 2014	Policy
3	Government of Nepal, Ministry of Urban Development	2014	National Water Supply and Sanitation Policy 2014 DRAFT	Policy
4	Government of Nepal, Ministry of Health	2015	National adolescent health and development strategy 2015	Strategy
5	Government of Nepal, Ministry of Health and Population	2015	Nepal Health Sector Strategy 2015–2020	Strategy
6	Government of Nepal, Ministry of Education	2016	School sector development plan 2016/17–2022/23	Strategy
7	Nepal Fertility Care Center	2017	Integrating MHH in school health program	Training
8	Government of Nepal, Ministry of Water Supply and Sanitation Sector Efficiency Improvement Unit	2017	Nepal Water Supply, Sanitation and Hygiene Sector Development Plan (2017–2030)	Strategy
9	Government of Nepal, Ministry of Education, Centre for education and human resource development	2018	School WASH procedure	Guidelines
10	Government of Nepal	2020	राष्ट्रिय मयााददत मष्ट्रहनावारी नीष्ट्रत २०७४ [MHH policy draft – google translate]	Policy

#### Data extraction and analysis

The EquiFrame is a systematic policy analysis framework, which evaluates the extent to which 21 core concepts of human rights and 12 vulnerable groups are included in health policy documents [30]. Each core concept of human rights has key questions and key language to support consistent understanding and scoring against policy content. For instance, the key question for the core concept, *Individualised services* is: ‘does the policy support the rights of vulnerable groups with individually tailored services to meet their needs and choices?’; its key language is: ‘vulnerable groups receive appropriate, effective, and understandable services’ [31]. Each reference to a core concept is awarded a quality of commitment score of 1 to 4: 1 = concept only mentioned; 2 = concept mentioned and explained; 3 = specific policy actions identified to address the concept; 4 = intention to monitor concept was expressed.

As our study focused on disability and gender, we adapted the EquiFrame tool to only focus on the inclusion of rights with regards to these two groups. We also refined the 21 core concepts of human rights key questions and key language to reflect WASH and MHM (see Additional files 1 and 2). We applied the original EquiFrame quality of commitment score continuum of 1 to 4. We also consulted the authors of the EquiFrame application manual, and they reviewed its content and endorsed its adaptation.

Guidance was developed for the review and scoring process to ensure consistency. To establish validity and reliability of the data, JW and NS independently assessed the content of each policy document for the inclusion of information relevant to the 21 core concepts, and scored each reference. Any differences were discussed before agreeing final scores. Data was captured across disability and gender, WASH and MHM for all materials.

Each reference that scored 3 or 4, was deemed ‘high quality’, as the information provided specific actions points and intention to monitor. For instance, the following text from the School Sector Development Plan referenced *Capability based services, Integration* and *Efficiency* against gender, WASH and MHM. Each reference to these core concepts were awarded 3 because specific actions are identified.

*“Nominate WASH focal teachers and menstrual hygiene management female teachers in all schools, for coordinating, planning, resource mobilisation, and monitoring of school WASH activities and facilities in coordination with school WASH coordination committees”* [32].

The percentage of the 21 core concepts referenced at least once, and the average score of each reference were presented for each document. To estimate the overall quality of commitments made to human rights, data was then aggregated across all documents to show the average score and total proportion of references made to each core concept.

### Qualitative study: materials and methods

#### Study site

Nepal was selected as the study focus due to the widely documented sociocultural restrictions and taboos surrounding menstruation and because the Government of Nepal is committed to improving gender equality in WASH [33–36]. Kathmandu and the Kavre District were selected as the study sites for the qualitative interviews. Kathmandu was selected as it is the administrative capital of Nepal, and the Kavre district was selected because it was already chosen as the Disabling Menstrual Hygiene Barriers research study site [23]. Kavre is one of Nepal’s 77 districts, with a population of 381,937, and estimated basic water coverage is 89%, whilst basic sanitation coverage is 98% [37] (water and sanitation coverage is from unpublished data). Ethical approval for the study was granted by from the Research Ethics Committee and the Health Research Council of the authors’ institutes.

#### Study population and sample size

The study population and inclusion criteria comprised: 12 key informants, who were government officials or service providers, working at the national level in Kathmandu or at the district level, in the Kavre District, with a professional focus on WASH, MHM, health, education or disability (Table 3). Topics explored were: involvement of civil society, and people with disabilities in policy discourse, development and implementation; mechanisms to monitor the implementation of policy commitments; knowledge of WASH and MHM requirements of people with disabilities, organisational focus on MHM for people with and without a disability, and support and guidance for service providers to deliver inclusive MHM interventions.

**Table 3:** Study population characteristics

Study population	Characteristics	*n* = 12
Government official	Department of Water Supply and Sewerage, Kathmandu	1
	Department of Education, Kathmandu	1
	Women, Children and Social Welfare, Kavre district	1
	Federal Affairs and Local Governance, Kavre district	1
Service provider	Non-Government Organisation, Water, sanitation and hygiene, including MHH, Kathmandu	1
	Non-Government Organisation, Sexual and reproductive health, including MHH, Kathmandu	1
	Organisation for Persons with Disabilities, Disability, Kathmandu	1
	Healthcare provider, Health, Kavre district	4
	Social mobiliser, Kavre district	1

We applied stratified purposive sampling to government officials and service providers as we believed these constituted a fairly homogeneous sample [38]. Key informants were selected through WaterAid Nepal's networks, and snowball sampling was applied to increase the sample (whereby participants identified other people to interview) [39, 40].

#### Data collection methods and analysis

Written informed consent was obtained and witnessed from each participant before enrolment. An information sheet and consent form (in English or Nepali) was given to, or read out to the participants by the research team. Informed written consent was received from all participants.

In-depth interviews were undertaken at the participant’s place of work and lasted up to 1 hour. With consent, interviews were conducted in person by the research team, in English or Nepali and were recorded on a voice recorder. Field notes were made after the interviews. The research team met at the end of each day to discuss potential bias in data collection and analyse the findings. Once data collection was complete, voice recordings were translated into English (where necessary) and transcribed. Transcriptions were checked for precision by Nepali research team members and WaterAid Nepal staff.

Data generated through interviews was analysed thematically, firstly against the adapted socio-ecological framework for MHM, which were then coded into smaller units based on the specific topics explored and experiences of participants [9]. Codes were developed iteratively: they were compared and connections between them were identified and analysed using NVivo 11. Co-authors reviewed the themes and analyses to ensure reliability; any differences were discussed before consensus of opinion was reached. Additionally, research findings were presented for discussion and validation with participants and key stakeholders at a meeting in Kathmandu.

#### Research team and training

Our all-woman qualitative research team consisted of the lead author, a Nepali Research Coordinator, and two Nepali Field Researchers who have a disability. The research team undertook a week-long training, led by the lead author and a LSHTM colleague on how to conduct ethical research on MHM with key informants, people who have a disability and their caregivers [23].

## Results

### The government of Nepal demonstrates high level commitment to progressively realising the right to water and sanitation for all its citizens

The Government of Nepal moved to federalism in 2015, to enable the division of powers between three independent tiers of government: Federal Ministries, Provincial Ministries and Local Governments [41]. Within the WASH sector the Federal Ministries are responsible for large infrastructure and revenue distribution to three tiers from the treasury; the Provincial Ministries are responsible for medium infrastructure and grants to the Local Government; and the Local Governments (rural and urban) ensure WASH services, small infrastructure and spend local revenue [41].

The Constitution of Nepal sets out ambitions to be an inclusive state, which guarantees that all citizens have the right to equality, and the state is committed to Gender, Equality and Social Inclusion (GESI) within poverty reduction [42]. Therefore, policies take account of existing power relations (e.g. between women and men, or social groups), and how these influence access to resources and participation in decision-making mechanisms. Nepal also voted in favour of the United Nations Resolution on the human right to water and sanitation [43]. Within WASH policies, gender, caste and ethnic groups, poverty, remoteness and disability are identified as the key issues in the GESI commitment [44].

Since the Sustainable Development Goals were set in 2015 (which includes hygiene within Target 6.2 under Goal 6: clean water and sanitation), progress using a multi-sectoral response to MHM has gained momentum [45]. For instance, in 2015 a training package to integrate menstrual hygiene into school health programmes was developed [46]; in 2016, the Water Supply Sanitation and Hygiene Sector Development Plan was launched which includes policy statements related to MHM [44]; and in 2017 the Ministry of Water Supply and Sanitation formed a taskforce of government agencies and civil society organisations (with representation across disability, WASH and health) to draft a national policy on menstruation. Additionally, the WASH in Schools Procedure includes a ranking to measure MHM standards across different schools [47]. Nepal’s Water Supply, Sanitation and Hygiene Sector vision is ‘improved public health and living standard of people of Nepal through safe, sufficient, accessible, acceptable, and affordable water, sanitation and hygiene services–any time, everyone and everywhere’ [44].

### References made to core concepts of human rights across all policies and supporting documents

Ten policies, strategies, guidance and training documents were reviewed. Two of these did not include any reference to the core concepts of human rights for people with disabilities, and women and girls in relation to WASH: The National Adolescent Health and Development Strategy and the National Health Sector Strategy [48, 49].

Table 4 presents the frequency that each core concept from the adapted EquiFrame was referenced across the remaining eight documents (total references %) and the quality of those references (average score), separately for disability and gender, WASH and MHM.

**Table 4:** Frequency of references to cover concepts of human rights and average scores across all documents, and the comparison between disability and gender

Core concepts	Disability				Gender				Proportion of total references to disability and gender (*n* = 274)	
	WASH (***n*** = 33 references)		MHM within WASH (***n*** = 2/33 references)		WASH (***n*** = 241 references)		MHM within WASH (***n*** = 148/241 references)			
	Total references (%)	Average score	Total references (%)	Average score	Total references (%)	Average score	Total references (%)	Average score	Disability total references (%)	Gender total references (%)
Non-discrimination	15%	2.2	0%	0	11%	2.3	8%	2.3	2%	10%
Individualised services	24%	3.0	50%	3.0	19%	2.9	26%	2.8	3%	17%
Entitlement/affordability	0%	0	0%	0	3%	2.9	3%	2.8	0%	3%
Capability based services	0%	0	0%	0	6%	3.0	2%	3.3	0%	5%
Participation	0%	0	0%	0	5%	2.2	0%	0	0%	4%
Coordination of services	0%	0	0%	0	0%	0	0%	0	0%	0%
Protection from harm	12%	2.8	0%	0	10%	2.8	9%	2.6	1%	9%
Liberty	0%	0	0%	0	0%	0	0%	0	0%	0%
Autonomy	0%	0	0%	0	1%	3.0	1%	4.0	0%	1%
Privacy	0%	0	0%	0	1%	2.0	1%	2.0	0%	1%
Integration	6%	3.0	50%	4.0	3%	3.1	4%	3.3	1%	3%
Contribution	0%	0	0%	0	0%	0	0%	0	0%	0%
Family resource	0%	0	0%	0	0%	0	1%	3.0	0%	0%
Family support	0%	0	0%	0	1%	1.0	0%	0	0%	1%
Cultural responsiveness	0%	0	0%	0	0%	2.0	1%	2.0	0%	0%
Accountability	0%	0	0%	0	0%	0	0%	0	0%	0%
Prevention	0%	0	0%	0	5%	2.1	9%	2.2	0%	5%
Capacity building	6%	3.0	0%	0	7%	3.1	8%	3.2	1%	6%
Access	36%	2.9	0%	0	20%	3.0	18%	3.1	4%	17%
Quality	0%	0	0%	0	4%	3.2	7%	3.2	0%	4%
Efficiency	0%	0	0%	0	3%	3.1	3%	2.5	0%	3%
Totals	**100%**		**100%**		**100%**		**100%**		**12%**	**88%**

**Fig. 1:**
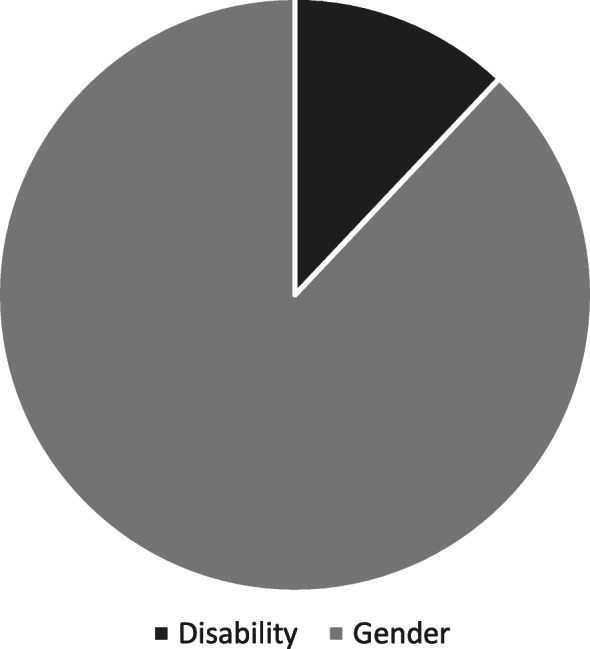
Number of references to core concepts of human rights related to disability and gender

**Fig. 2:**
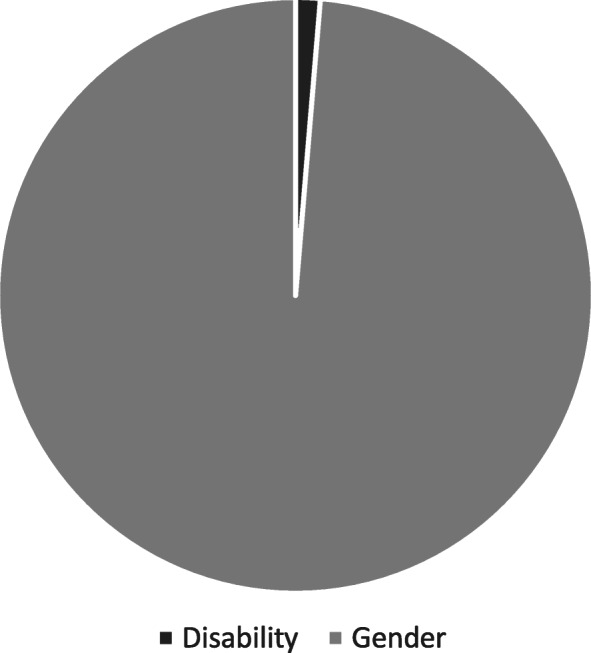
Number of references to core concepts of human rights against disability and gender, within MHM

Out of 274 references to either gender or disability across all of the materials, 88% were related to gender and 12% were related to disability (Fig. 1). Specifically, within the 148 references to MHM, 99% were gender-related and 1% (*n* = 2) were disability related (Fig. 2). Disability was referenced to 6 of the 21 core concepts of human rights, compared with 15 for gender. Across disability and gender, *Access* was most frequently mentioned (36 and 20% respectively). Highest quality references across all results were for *Integration* (average score 3.4) and *Individualised Services* (2.9). *Family support* received the lowest average score [2], meaning that overall, references to this core concept were not assigned specific policy actions or monitoring mechanisms.

Findings from the key informant interviews show that mechanisms are in place to ensure GESI in implementation of policies at the national and district level, including allocating budget from the national to district level to interventions targeting groups that may be marginalised. However, government officials at both the national and district level noted that policy implementation was weak. Reasons include: a lack of monitoring, inadequate staffing levels and budget allocation for inclusive WASH and MHM infrastructure; limited disability service providers, absence of training on MHM and disability and partial understanding of disability.

#### Limited involvement of people with disabilities in policy discourse and development

The core concepts, *Participation* and *Capability based services* (the latter recognizes the capabilities of people with disabilities in implementation and management of WASH activities at an organisational level) were referenced against gender (5 and 6% respectively), but they were not referenced against disability in any document.

*“Women’s participation will be emphasized in all aspects of water supply and sanitation planning, implementation, management, operation and maintenance.”* [50] (page 12; scored *Capability based services, Participation* against gender).

Key informants stated that Organisations of Persons with Disabilities (OPDs) and disability service providers must actively be involved in policy discourse and development to ensure that GESI is included in national policies. Policy makers explained that it is ‘compulsory’ to incorporate disability in every policy.

“The Disability and Gender terminologies cannot be excluded while developing any policy. It is a national commitment, that we must include them” (National government official).

However, the involvement of people with disabilities in policy discourse and development was not enforced, and participants expressed concerns that the involvement of people with disabilities could be tokenistic.

“ … unfortunately they are there because they were invited because someone thought it’s important to see it” (Service provider, Kathmandu).

National level government officials reported that advice was sought from disability advisors (who did not have a disability) from within the relevant ministry. Reasons given for the lack of involvement of people with disabilities and OPDs in policy development included the protracted process of getting policies and guidelines signed off by the government, even without involving external groups.

#### Inadequate policy monitoring, limited capacities and resources to implement policies at the district level

Across all documents reviewed *Access* (supports people with disabilities with physical and information access to WASH services) was most frequently mentioned (36%) against disability, and this was followed by *Individualised services* (24%) (which support the rights of people with disabilities with individually tailored WASH services to meet their needs, choices and impairments). Together these make up more than half (61%) of all references made to disability. Similarly for gender, *Access* receives nearly one quarter of all references (20%).

*“For all schools to have functional water and sanitation facilities that are environmentally sound and user-friendly for children, boys and girls and differently-abled students and teachers.”* [32] (page 63, scored *Access* and *Individualised services* against disability and gender).

With respect to disability and MHM, *Individualised services* and *Integration* were the only core concepts referenced.

*“Provision of adequate and CGD-friendly [accessible] WASH services including hand washing with soap and MHM facilities.”* [44] (page 39; scored *Individualised services* against disability and gender for WASH and MHM).

*“The National Institute of Education shall make provision for the inclusion of the term of Menstruation in an inclusive manner and include it in government and non-government schools and non-formal education.”* [29] (scored *Integration* against disability and gender for WASH and MHM).

However, these commitments were not realised: key informants reported that school latrines were not fully accessible and did not include incinerators to dispose of menstrual hygiene materials (incinerators were explicitly mentioned by the key informant as the expected disposal option). Reasons cited included inadequate budget allocation per unit, insufficient technical training for service providers and limited technical skills.

“The implementation is not that successful. [ ….] One of the problem was insufficient budget per unit. If you go to schools, you can see ramps but the ramps aren’t wide enough, those are because of lack of experience and technical problems. [ ….]” (Service provider, Kavre district).

*Accountability* was not referenced at all against disability or gender in any policy or supporting document. However, national government officials reported that disability rights groups were involved in monitoring policy implementation at the district level, but district government officials explained that this did not happen. They also described inadequate staffing levels within the district government, and feeling over stretched by commitments across different sectors.

“…. proper manpower should be allocated in the VDC’s [Village Development Committees] and Municipalities for formulating and implementing and monitoring of the policies. Also, the people working in the government offices should also be allocated specific sectors, a person cannot work in all sectors and look after all sectors. So, the policies and regulations are very weak” (District level government official).

#### Limited disability service providers and outreach for people with disabilities at the district level

The core concept, *Coordination of services* was not referenced at all in any policy or supporting document in relation to disability or gender. Participants explained that MHM programmes in Kavre were delivered collaboratively by NGOs (including disability service providers), district government officials and healthcare workers. Government funds could be applied for by implementing organisations, but district government officials noted these were not often sought or applied for, partly because groups focussed on disability-inclusive development were not particularly active in the Kavre district, though they did exist.

“This financial year we were not able to work on any programs related to disabled people because [ ….] the disabled people cannot make their own program, and the team responsible for developing programs for the disabled people were not active” (District level government official).

#### A lack of data on MHM barriers faced by people with disabilities leads to limited professional training and support to address the issues

As part of commitments to GESI, training on MHM was delivered at the national and district levels by NGOs, which shows how MHM is becoming a more significant political priority in Nepal. At the national level, policy makers were involved in ‘master trainer of trainer’ programmes for MHM, in which people with disabilities were present. However, this training did not include awareness of the barriers to MHM that people with disabilities face. This disparity is reflected in the documents reviewed: with respect to MHM, *Capacity building* received 8% of references against gender, whilst it was not referenced at all in relation to disability. Furthermore, *Quality* (which highlights the need for evidence-based and professionally skilled practice) received 7% of references against gender, but was not cited against disability in any of the documents.

Among the key informants, there was a better understanding of the barriers to WASH facilities for people with mobility limitations than other impairment types.

“There hasn’t been much work done for the menstrual hygiene management of disabled children. We have a provision for separate toilets for boys and girls, and about the disabled friendly toilets, there are ramps in the toilets” (National level government official).

Service providers, who were aware that disability needs to be integrated into MHM interventions, did not have the knowledge or tools to be able to support people with disabilities and their caregivers. One expressed frustration at their inability to do so.

“You know [people with intellectual impairments] take the pad and put it on their face. I just don’t have an answer. It makes me feel very bad, but I don’t have an answer. I have no idea about what to do about that. And there’s so little information that’s accessible to understand what to do” (Service provider, Kathmandu).

#### Limited professional training and support leads to inadequate understanding of the issues

The national government official who completed the MHM awareness raising training for policy makers and service providers, which was attended by people with disabilities, noted that the interactions with these participants increased their awareness of disability, but that limited understanding of disability in Nepal is pervasive.

“.. in Nepal there is poor understanding about people with disability. There is common understanding that people having problem with legs and limbs, or paralyzed people, they are treated as people with disabilities, but in my opinion people with visual or hearing impairment, people with backbone problems are not included in the disability groups” (National level government official).

A limited understanding of disability was clear through interviews with key informants in the Kavre district. For instance, when a healthcare worker was asked if they knew of any people with disabilities living in the district, they replied that there is one girl who has a physical impairment as well as: “a 11-12-year-old girl who has problems with her earlobes” (Service provider, Kavre district). One service provider in the Kavre district explained: “I don’t have much knowledge on menstrual hygiene problems of disabled people.”

#### People with disabilities are marginalised within MHM interventions

District health care workers received MHM training from NGOs; they then trained health assistants and school teachers to teach school children to make menstrual materials, and dispose or reuse them hygienically. However, the training did not include disability and participants noted that very few children with a disability attended schools. Even though *Non-discrimination* was referenced 15% against disability across all documents, and *Integration* was one of the two core concepts referenced against disability with respect to MHM, there were no MHM outreach programmes for children with disabilities who were not in school. Reasons given by participants for this lack of representation were that children with disabilities did not attend those schools, and that people with disabilities were unable to travel to meeting locations. The core concept, *Entitlement/affordability* was not referenced against disability in any document.

Researcher: Were there children with disabilities in the [MHM training] programmes?

Participant: No. Because most of the participants were girls from the schools in our ward, and there were no disabled girls in those schools. We don’t know much about disabled girls because they can’t be present themselves in all the programs (Service provider, Kavre district).

*Protection against harm* was referenced 12% with respect to disability in the documents reviewed, but three service providers reported being approached regularly by family members of people with disabilities seeking to cease menstruation.

Researcher: Have you ever been asked about sterilization?

Participant: Yes. That is the one of the often question the parents ask me. The caretaker and parents want disabled women to get surgical sterilization so that there is no problem of taking care of menstruation and no risk of unwanted pregnancy (Service provider, Kathmandu).

Medical records on sterilisation of people with disabilities were sought, but could not be obtained. Service providers in Kavre reported that caregivers felt that menstruation was pointless for people with disabilities as they will never be married, or be able to understand how to manage menstruation. One service provider expressed agreement.

“First, most of the disabled girls don’t know about menstruation, they don’t know [what] menstruation is, what hygiene is. They even take out the sanitary pads their parents put on them. So, for the mentally disabled girls, menstruation doesn’t make sense for them” (Service provider, Kavre district).

The value-based language used by the participants is of particular note: “so that there is no problem of taking care of menstruation”, and “menstruation doesn’t make sense for them”. *Autonomy* (supports the right of people with disabilities to exercise choice or control of what happens to them), *Family resource* and *Family support* (recognizes individual members of people with disabilities may have an impact on the family members requiring additional support from WASH services) are not referenced at all in relation to disability in any of the documents reviewed.

## Discussion

This study aimed to review the extent to which the needs of people with disabilities are included in Nepal’s policies and supporting documents related to MHM and WASH, and explores how these policy commitments are implemented, using the Kavre district as a case study. The Government of Nepal is committed to progressively realising the right to water and sanitation for all its citizens, demonstrated through its national level commitment to GESI. However, disability rights receive inadequate attention in policies and supporting documents compared to gender. Within WASH, MHM policy commitments for people with disabilities is almost non-existent. This translates into practice: people with disabilities are marginalised within MHM interventions.

Existing evidence demonstrates that a dearth of data about the barriers to MHM that people with disabilities and their caregivers face, leads to a lack of awareness, understanding, support and guidance for MHM and disability [9, 51]. Our study reflects these patterns, and this can be tracked through an absence of: 1) rights for people with disabilities with regards to MHM in national policies and supporting documents, 2) professional training and support for service providers, and 3) professional understanding of the issues.

Our policy analysis of relevant documents reveals that, when compared to gender, disability is excluded in national policies and supporting documents. Additionally, a more holistic strategy to improving gender equality (which includes promoting women’s leadership and redressing gender power inequalities) is applied within Nepal’s commitment to GESI. For instance, when comparing references of core concepts of human rights between disability and gender in policies and supporting documents: gender receives 88% of references across a much broader spread of commitments to core concepts than is seen for disability. Additionally, there are only two references made to disability and MHM in all documents, compared to 148 for gender and MHM. Therefore, the impact of not having their MHM needs addressed may compound the double discrimination experienced by women and girls with disabilities compared to boys and men with disabilities.

An assumption could be made that if MHM is included within a policy document, then gender equality is addressed. However, meeting the needs of women and girls who menstruate is an important step towards gender equality, but it is only a component of it. Identifying which of the core concepts within the EquiFrame are referenced provides a valuable tool for interrogating strengths and gaps in policy documentation. For instance, transformative gender approaches include ensuring women and girls receive specific, appropriate and effective MHM services, with reasonable adjustments made/supported, when necessary (*Individualised Services)*, but also that women and girls hold leadership positions with decision making responsibilities, and they are supported to perform these roles if required (*Capability Based Services*); government structures support sex and age disaggregated data (*Quality*)*,* and hygiene facilities, goods and services are respectful of ethical principles and culturally appropriate (*Cultural responsiveness*). Therefore, gender equality requires references to a broad spread of core concepts of human rights. This is mirrored in disability inclusion. It is not enough to ensure MHM facilities are accessible for people with disabilities (*Individualised services*); agency must be enhanced to ensure people with disabilities take decisions in relation to menstrual hygiene and that these decisions are acted upon (*Participation* and *Autonomy*). Key informants noted that the participation of people with disabilities in policy discourse and development were expected, but not enforced and was tokenistic. A lack of political engagement by people with disabilities is well referenced in global literature, even though it is a core human rights principle within the Convention on the Rights of Persons with Disabilities [16, 52–56].

In terms of policy implementation, challenges that exist in the Kavre district include: inadequate staffing capacities and allocation of resources to implement and monitor national commitments, even where policy document references to core concepts are scored as ‘high quality’ and provide specific action points (i.e. the School Sector Development Plan). Reasons given were inadequate budget allocation for infrastructure, technical training and professional skills. This is unsurprising as limited capacities to implement national financing plans for WASH is a well-documented globally. In 2019, 75% of 107 countries reported having financing plans for WASH, but more than half of these were not implemented effectively, and less than 15% of countries had the required human resources or funds to deliver WASH plans [56]. In terms of monitoring, 79% of countries have government led monitoring processes of national WASH targets, but only 10% reported having the human resources to do this [56].

In the Kavre district, disability service providers had limited capacities and did not access all government funds allocated for disability programmes. A lack of ability to spend funds could result in even less focus on disability in national policies and plans. Our findings support existing literature on disability service provision, which highlight successes, but also challenges related to a lack of financial commitments, professional understanding and training as major challenges to implementation [57–60].

Supporting the development of evidence-based and professionally skilled practice *(Quality)* was not prioritised in policies and guidance documents. This resulted in key informant’s limited understanding of disability and a focus on the requirements of people with mobility impairments. People with intellectual impairments were mentioned in relation to an inability to support their MHM requirements, and there was no mention of people with visual, hearing or communication impairments. People with these impairments are harder to reach through broad based interventions as additional inclusion measures are needed, such as sign language interpretation, working with caregivers or providing clear, simple and repetitive information [16]. Subsequently, a WASH infrastructure-based approach to improving MHM for people with mobility impairments in Nepal is prioritised, instead of a comprehensive and holistic strategy that addresses every aspect of MHM for all impairment groups, such as the provision of accurate and accessible information on the menstrual cycle and how to manage it hygienically.

Key informants cited successes related to accessible school toilets, but supporting children with disabilities in accessing school to use the facilities, and communicating MHM information in an accessible way were completely absent, even though this was committed to in the documents reviewed. None of the MHM interventions in schools included children with disabilities. Therefore, people with disabilities are marginalised within MHM interventions. As defined by de Albuquerque, marginalisation ‘refers to the process that systematically denies people opportunities and resources that are available to other members of society, and which would otherwise serve to promote social integration’ [61]. Furthermore, as people with disabilities could not access information and services related to reproductive health, their right to health is violated. Existing literature calls for the integration of MHM into the education system, with efforts to ensure that these are inclusive of disability [4, 62]. However, efforts must go further - outreach programmes, for people with disabilities who are not in school, must complement MHM programmes delivered through school platforms.

Global literature on MHM for people with disabilities who are unable to manage menstruation independently, highlights the central role of caregivers, as well as a lack of MHM information, guidance and support for them [11, 12, 14, 15, 63]. Our study reflects this. Within policy documents, the human rights core concepts, *Family resource* (which recognizes the value of the family members of people with disabilities in addressing MHM needs), *Family support* and *Autonomy*, were not referenced against disability. There was also inadequate support and MHM guidance for caregivers in the Kavre district, which can lead caregivers to feel overwhelmed and isolated [23]. These factors, along with menstrual taboos and disability discrimination, can result in the sterilisation of the person with a disability in order to cease menstruation and guard against unwanted pregnancies [11, 12, 51, 64–66]. We found indications that caregivers seek to sterilise people with disabilities who are unable to manage their menstruation independently. We also found that ‘The National Adolescent Health’ and ‘Development Strategy’ and the ‘Nepal Health Sector Strategy’ did not consider MHM, which indicates that MHM is not consistently considered part of health, or specifically sexual and reproductive health [48, 49]. A cross sectoral approach to addressing the sexual and reproductive health and rights of people with disabilities, and women generally, needs to be considerably strengthened.

### Study strengths and limitations

A key strength of this study is that we used a structured tool for policy analysis to assess the inclusion of disability, compared to the inclusion of gender, in relevant policies and supporting documents in Nepal. We complemented this by looking at one district to consider the implementation of policies around disability, WASH and MHM.

Some limitations should be considered when interpreting the study findings. These include potential selection bias during the identification of policies and supporting documents, and that the MHM policy draft [29] was translated from Nepali into English via Google Translate. The latter was managed by a Nepali speaker who checked the translation and corrected inaccuracies. Expired policies and supporting documents were not included so an analysis of shifting policy priorities over time was not conducted.

## Conclusion

Though the Constitution of Nepal enshrines disability inclusion, we highlight consistent gaps in attention to disability and MHM in policies and practice. The Government of Nepal should invest in generating rigorous evidence about the barriers to MHM that people with disabilities and their caregivers face. Drawing on this data, training on these issues should be developed and delivered to improve professional understanding. With a more nuanced knowledge, policy makers could then integrate more core concepts of human rights against disability in policies and supporting documents and increase the focus on MHM. A more comprehensive, holistic and cross-sectoral strategy must be developed to addresses every aspect of MHM for people with disabilities, and will require provision of support and guidance for service providers. Addressing these recommendations would allow the Government of Nepal to progressively realise the rights of persons with disabilities to water and sanitation.

## Supplementary Information

**Additional file 1.** Adapted EquiFrame for WASH, MHM and Disability.

**Additional file 2.** Adapted EquiFrame for WASH, MHM and Gender.

## Data Availability

The data underlying this article will be shared on reasonable request to the corresponding author.

## Abbreviations

GESI: Gender, Equality and Social Inclusion
LMIC: Low- and middle-income country
*[LSHTM]*: *[London School of Hygiene & Tropical Mecidine]*
MHM: Menstrual Health and Hygiene
MHM: Menstrual Hygiene Management
NGO: Non-Government Organisation
OPD: Organisations of Persons with Disabilities
VDC: Village Development Committees
WASH: Water, sanitation and hygiene
