# Supplementary material for: Are Nepal’s water, sanitation and hygiene and menstrual hygiene policies and supporting documents inclusive of disability? A policy analysis
Source: Int J Equity Health. 2021 Jul 8;20:157. doi: 10.1186/s12939-021-01463-w (PMC8268379; doi:10.1186/s12939-021-01463-w)
Supplement: Supplementary file 1 — Additional file 1. Adapted EquiFrame for WASH, MHM and Disability. [file 12939_2021_1463_MOESM1_ESM.docx]

**Additional File 1. Adapted EquiFrame for WASH, MHM and Disability**

| Core concept | Disability & WASH | | Disability & MHM | |
| --- | --- | --- | --- | --- |
|  | **Key question** | **Key language** | **Key question** | **Key language** |
| Non-  discrimination | Does the policy support the rights of people with disabilities with equal opportunity in accessing WASH services? | People with disabilities are not directly or indirectly discriminated against within the WASH system. | Does the policy support the rights of people with disabilities with equal opportunity in accessing MHM services? | People with disabilities are not directly or indirectly discriminated against within the MHM system. |
| Individualised  services | Does the policy support the rights of people with disabilities with individually tailored WASH services to meet their needs, choices and impairments? | People with disabilities receive specific, appropriate and effective WASH services, with reasonable adjustments made/supported, when necessary. | Does the policy support the rights of people with disabilities with individually tailored MHM services to meet their needs, choices and impairments? | People with disabilities receive specific, appropriate and effective MHM services, with reasonable adjustments made/supported, when necessary. Including:  1. Clean menstrual materials  2. Private and accessible place to change these materials (e.g. toilet) 3. Accessible soap and water for washing the body  4. Safe, accessible and convenient facilities to dispose of used materials  5. Accessible, accurate information on the menstrual cycle |
| Entitlement/  affordability | Does the policy indicate entitlements for people with disabilities (e.g. respite grant or reduced user fee) and how they may qualify for specific benefits relevant to them? | People with disabilities who have limited resources are entitled to some services free of charge or may be entitled to a sliding scale tariff. | Does the policy indicate entitlements for people with disabilities (e.g. respite grant, reduced fee or no tax for menstrual products) and how they may qualify for specific benefits relevant to them? | People with disabilities who have limited resources are entitled to menstrual products free of charge or at a reduced cost. |
| Capability  based services | Does the policy recognize the capabilities of people with disabilities in implementation and management of WASH activities, at an organisational level? | For instance, peer support, group advocacy, DPOs. | Does the policy recognize the capabilities of people with disabilities in relation to MHM? | For instance, peer support, group advocacy, DPOs focused on MHM. People with disabilities are meaningfully represented in MHM management committees |
| Participation | Does the policy support the right of people with disabilities to participate in the decisions that affect their lives and enhance their empowerment? | People with disabilities can exercise choices and influence decisions affecting their life. They are included and consulted in the planning, development, implementation, and evaluation of WASH activities | Does the policy support the right of people with disabilities to participate in the decisions related to MHM that affect their lives and enhance their empowerment? | People with disabilities can exercise choices and influence decisions affecting their life. Such consultation may include planning, development, implementation, and evaluation related to MHM. |
| Coordination  of services | Does the policy support assistance of people with disabilities in accessing services from within a single provider system (interagency/utility) or more than one provider system (intra-agency/utility) or more than one sector (inter-sectoral)? | People with disabilities know how services should interact where inter-agency, intra-agency, and inter-sectoral collaboration is required. This includes coordination between health services, schools, households and public places, with regards to WASH. Additional coordination opportunities include WASH sector with the private sector, civil society and rights groups. | Does the policy support assistance of people with disabilities in accessing MHM services from within a single provider system (interagency/utility) or more than one provider system (intra-agency/utility) or more than one sector (inter-sectoral)? | People with disabilities know how MHM services should interact where inter-agency, intra-agency, and inter-sectoral collaboration is required. This includes coordination between health services, schools, households and public places, with regards to MHM. Additional coordination opportunities include WASH / MHM actors with the private sector, civil society and rights groups. |
| Protection  from harm | People with disabilities are protected from harm during their interaction with WASH and related services | People with disabilities are protected from harm during their interaction with WASH services, as well as health related system and from families and the community who may have negative attitudes about WASH for people with disabilities. This includes the right to private, secure WASH facilities. | People with disabilities are protected from harm during menstruation and related services | People with disabilities are protected from harm during menstruation and their interaction with related services, as well as health related system and from families and the community who may have negative attitudes about menstruation and people with disabilities. |
| Liberty | Does the policy support the right of people with disabilities to be free from unwarranted physical or other confinement? | People with disabilities are protected from unwarranted physical or other confinement while in the custody of the service system/provider. This includes at home and a healthcare service. | Does the policy support the right of people with disabilities to be free from unwarranted physical or other confinement during menstruation? | People with disabilities are protected from unwarranted physical or other confinement during menstruation. This includes at home, boarding schools and a healthcare service. |
| Autonomy | Does the policy support the right of people with disabilities to consent, refuse to consent, withdraw consent, or otherwise control or exercise choice or control over what happens to her or him? | People with disabilities can express “independence” or “self-determination”. For instance, person with an intellectual disability will have recourse to an independent third party regarding issues of consent and choice. | Does the policy support the right of people with disabilities to consent, refuse to consent, withdraw consent, or otherwise control or exercise choice or control over what happens to them when menstruating? | People with disabilities can express “independence” or “self-determination” in relation to menstruation. For instance, person with an intellectual disability will have recourse to an independent third party regarding issues of consent and choice over the menstrual product used, method to manage menstrual discomfort. |
| Privacy | Does the policy address the need for information regarding people with disabilities to be kept private and confidential? | Information regarding people with disabilities need not be shared among others. | Does the policy address the need for information regarding people with disabilities who menstruate to be kept private and confidential? | Information regarding people with disabilities who menstruate need not be shared among others. |
| Integration | Does the policy promote the use of mainstream services by people with disabilities? | People with disabilities are supported to use the services that are provided for general population. | Does the policy promote the use of mainstream MHM services by people with disabilities? | People with disabilities are supported to use the MHM services that are provided for general population. |
| Contribution | Does the policy recognize that people with disabilities can be productive contributors to society and the WASH sector? | People with disabilities make a meaningful contribution to society and the WASH sector. They are recognised as important contributors to programmes and activities | Does the policy recognize that people with disabilities can be productive contributors to society during menstruation? | People with disabilities who are menstruating make a meaningful contribution to society. |
| Family  resource | Does the policy recognize the value of the family members of people with disabilities in addressing WASH needs? | The policy recognizes the value of all family members of people with disabilities, in supporting WASH needs and activities | Does the policy recognize the value of the family members of people with disabilities in addressing MHM needs? | The policy recognizes the value of family members of people with disabilities as a resource for addressing MHM needs. |
| Family  support | Does the policy recognize individual members of people with disabilities may have an impact on the family members requiring additional support from WASH services? | Caring for persons with disabilities may have mental health effects on other family members, such that these family members themselves require support. | Does the policy recognize individual members of people with disabilities may have an impact on the family members requiring additional support from MHM services? | Caring for persons with disabilities who menstruates may have mental health effects on other family members, such that these family members themselves require support. |
| Cultural responsiveness | Does the policy ensure that services respond to the beliefs, values, gender, interpersonal styles, attitudes, cultural, ethnic, or linguistic, aspects of the person, as well as personal safety and dignity? | i) People with disabilities are consulted on the acceptability of the service provided ii) Hygiene facilities, goods and services must be respectful of ethical principles and culturally appropriate, i.e. respectful of the culture of people with disabilities | Does the policy ensure that MHM services respond to the beliefs, values, gender, interpersonal styles, attitudes, cultural, ethnic, or linguistic, aspects of the person, as well as personal safety and dignity? | i) People with disabilities who menstruate are consulted on the acceptability of the MHM service provided ii) Hygiene facilities, goods and services must be respectful of ethical principles and culturally appropriate, i.e. respectful of the culture of people with disabilities |
| Accountability | Does the policy specify to whom, and for what, services providers are accountable? | People with disabilities have access to internal and independent professional evaluation or procedural safe guard. Do laws/regulations provide mechanisms that ensure complaints are effectively heard? Are there effective complaint mechanisms? Are there judicial bodies that can resolve conflicts? This is evident for both public and private institutions. | Does the policy specify to whom, and for what, MHM services providers are accountable? | People with disabilities have access to internal and independent professional evaluation or procedural safe guard related to MHM. Do laws/regulations provide mechanisms that ensure complaints regarding MHM are effectively heard? Are there effective complaint mechanisms? Are there judicial bodies that can resolve conflicts? This is evident for both public and private institutions. |
| Prevention | Does the policy support people with disabilities in seeking primary, secondary and tertiary prevention of health conditions associated with WASH? | Includes WASH related illnesses and details on how people with disabilities can seek primary, secondary and tertiary prevention of health conditions associated with WASH, e.g. Trachoma, Soil-Transmitted Helminthes – intestinal worms, Lymphatic Flariasis, Leprosy | Does the policy support people with disabilities in seeking primary, secondary and tertiary prevention of health conditions associated with menstruation? | Includes complications related to menstruation e.g. reproductive tract infections such as bacterial vaginosis, candidia, and Trichomonas vaginalis (including genital itching, back pain, abdominal pain, pustules over genitalia and abnormal genital discharge) |
| Capacity  building | Does the policy support the capacity building of health workers and of the system that they work in addressing WASH needs of people with disabilities? | Includes awareness raising among communities and families on disability and barriers faced by people with disabilities. | Does the policy support the capacity building of health workers and of the system that they work in addressing MHM needs of people with disabilities? | Includes awareness raising among communities and families on disability and barriers to MHM faced by people with disabilities. |
| Access | Does the policy support people with disabilities – physical and information access to WASH services? | People with disabilities have accessible and safe WASH services within, or in the immediate vicinity, of household, health and educational institution, public institutions and places and workplace. All information must be understandable and in appropriate format. | Does the policy support people with disabilities – physical and information access to MHM services? | People with disabilities have accessible and safe MHM facilities within, or in the immediate vicinity, of household, health and educational institution, public institutions and places and workplace. Includes: 1) Private and accessible place to change these materials (e.g. toilet), 2. Accessible soap and water for washing the body, 3. Safe, accessible and convenient facilities to dispose of used materials. All MHM information must be understandable and in appropriate format. |
| Quality | Does the policy support quality services to people with disabilities through highlighting the need for evidence-based and professionally skilled practice? Does the policy promote innovation in WASH services for people with disabilities (e.g. technology)? Does the policy support water that is safe for consumption and other personal uses, so that it presents no threat to human health for people with disabilities; that sanitation facilities must be hygienically and technically safe to use for people with disabilities and ensure hygiene, access to water for cleansing and hand washing for people with disabilities at critical times. | People with disabilities are assured of the quality of water supply for consumption and other personal uses; that sanitation facilities are hygienically and technically safe to use by people with disabilities and that water for hygiene (cleansing, handwashing) is accessible at critical times (i.e. during menstruation, after defecation, changing diapers/nappies, before preparing food and before eating). Services are based on best practice/evidence and support innovative strategies/technology. This includes guidelines. | Does the policy support quality MHM services to people with disabilities through highlighting the need for evidence-based and professionally skilled practice? Does the policy promote innovation in MHM services for people with disabilities? E.g. menstrual product type, information exchange, accessibility of water for washing the body/menstrual product used; safe, private and accessible place to change the menstrual product; accessible methods to dispose of the used menstrual product)? | People with disabilities are assured that disposal facilities are hygienically and technically safe to use by people with disabilities and that water for menstrual hygiene is accessible during menstruation. Services are based on best practice/evidence and support innovative strategies/technology |
| Efficiency | Does the policy support efficiency by providing a structured way of matching WASH system resources with service demands in addressing WASH needs of people with disabilities? | Are WASH services sustainable for people with disabilities: Will services still be available in times of financial crisis? Are technology choices appropriate? Do contracts with providers take into account operation and maintenance? Are funds from donors sustainable? | Does the policy support efficiency by providing a structured way of matching MHM system resources with service demands in addressing MHM needs of people with disabilities? | Are MHM services sustainable for people with disabilities: Will services still be available in times of financial crisis? Are technology choices and menstrual products appropriate? Do contracts with providers take into account operation and maintenance? Are funds from donors sustainable? |
