# Supplementary material for: Are Nepal’s water, sanitation and hygiene and menstrual hygiene policies and supporting documents inclusive of disability? A policy analysis
Source: Int J Equity Health. 2021 Jul 8;20:157. doi: 10.1186/s12939-021-01463-w (PMC8268379; doi:10.1186/s12939-021-01463-w)
Supplement: Supplementary file 2 — Additional file 2. Adapted EquiFrame for WASH, MHM and Gender. [file 12939_2021_1463_MOESM2_ESM.docx]

**Additional File 2. Adapted EquiFrame for WASH, MHM and Gender**

| Core concept | Gender & WASH | | Gender & MHM | |
| --- | --- | --- | --- | --- |
|  | **Key question** | **Key language** | **Key question** | **Key language** |
| Non-  discrimination | Does the policy support the rights of women/girls with equal opportunity in receiving WASH services? | Women/girls are not directly or indirectly discriminated against within the WASH system. | Does the policy support the rights of people with equal opportunity in receiving MHM services? | People (women/girls, LGBTI people) are not directly or indirectly discriminated against within the MHM system. |
| Individualised  services | Does the policy support the rights of women/girls with individually tailored WASH services to meet their needs and choices? | Women/girls receive specific, appropriate and effective WASH services, for example services specific to menstrual hygiene management | Does the policy support the rights of people who menstruate with individually tailored MHM services to meet their needs and choices? | People who menstruate receive specific, appropriate and effective MHM services, for example WASH services specific to menstrual hygiene management. Including:  1. Clean menstrual materials  2. Private place to change these materials (e.g. toilet) 3. Soap and water for washing the body  4. Safe and convenient facilities to dispose of used materials 5. Accurate information on the menstrual cycle |
| Entitlement/  affordability | Does the policy indicate entitlements for women/girls (e.g. respite grant or reduced user fee), and how they may qualify for specific benefits relevant to them? | Women/girls may be entitled to a respite grant or sliding scale tariff, especially if in unpaid work | Does the policy indicate entitlements for people who menstruate (e.g. respite grant, reduced fee or no tax for menstrual products) and how they may qualify for specific benefits relevant to them? | People who menstruate who have limited resources are entitled to menstrual products free of charge or at a reduced cost. |
| Capability  based services | Does the policy recognize the capabilities of women/girls in implementation and management of WASH activities, at an organisational level?? | For instance, peer support, mentoring, group advocacy. Women are meaningfully represented in WASH committees. | Does the policy recognize the capabilities of people who menstruate in relation to MHM? | For instance, peer support, mentoring, group advocacy focusing on MHM. |
| Participation | Does the policy support the right of women/girls to participate in the decisions that affect their lives and enhance their empowerment? | Women/girls can exercise choices and influence decisions affecting their life. They are included and consulted in the planning, development, implementation, and evaluation of WASH activities | Does the policy support the right of people who menstruate to participate in the decisions related to MHM that affect their lives and enhance their empowerment? | People who menstruate can exercise choices and influence decisions affecting their life. Such consultation may include planning, development, implementation, and evaluation related to MHM. |
| Coordination  of services | Does the policy support assistance of women/girls in accessing services from within a single provider system (interagency) or more than one provider system (intra-agency) or more than one sector (intersectoral)? | Women/girls know how services should interact where inter-agency, intra-agency, and inter-sectoral collaboration is required. This includes coordination between health services and schools with regards to WASH. Additional coordination opportunities include WASH sector with the private sector, civil society and rights groups. | Does the policy support assistance of people who menstruate in accessing MHM services from within a single provider system (interagency/utility) or more than one provider system (intra-agency/utility) or more than one sector (intersectoral)? | People who menstruate know how MHM services should interact where inter-agency, intra-agency, and inter-sectoral collaboration is required. This includes coordination between health services, schools, households and public places, with regards to MHM. Additional coordination opportunities include WASH / MHM actors with the private sector, civil society and rights groups. |
| Protection  from harm | Women/girls are protected from harm during their interaction with WASH and related services | Women/girls are protected from harm during their interaction with WASH services, as well as health related systems, including sexual violence. Also protection from harm from families and the community who may have negative attitudes about specific WASH topics for women/girls (e.g. menstrual hygiene). This includes the right to private, secure WASH facilities. | People who menstruate are protected from harm during menstruation and related services | People who menstruate are protected from harm during menstruation and their interaction with related services, as well as health related system and from families and the community who may have negative attitudes about menstruation. |
| Liberty | Does the policy support the right of women/girls to be free from unwarranted physical or other confinement? | Women/girls are protected from unwarranted physical or other confinement while in the custody of the service system/provider. This includes at home and a healthcare service. | Does the policy support the right of people who menstruate to be free from unwarranted physical or other confinement during menstruation? | People who menstruate are protected from unwarranted physical or other confinement during menstruation. This includes at home, boarding schools and a healthcare service. |
| Autonomy | Does the policy support the right of women/girls to consent, refuse to consent, withdraw consent, or otherwise control or exercise choice or control over what happens to her or him? | Women/girls can express “independence” or “self-determination”. For instance, a husband is not to make decisions for his wife. | Does the policy support the right of people who menstruate to consent, refuse to consent, withdraw consent, or otherwise control or exercise choice or control over what happens to them when menstruating? | Women/girls can express “independence” or “self-determination” in relation to menstruation. For instance, a wife is able to use the household budget to buy menstrual products or relief for menstrual discomfort. |
| Privacy | Does the policy address the need for information regarding women/girls to be kept private and confidential? | Information regarding women/girls need not be shared among others. | Does the policy address the need for information regarding people who menstruate to be kept private and confidential? | Information regarding people who menstruate need not be shared among others. |
| Integration | Does the policy promote the use of mainstream services by women/girls? | Women/girls are supported to use the services that are provided for general population. | Does the policy promote the use of mainstream services by people who menstruate? | People who menstruate are supported to use the services that are provided for general population. |
| Contribution | Does the policy recognize that women/girls can be productive contributors to society and the WASH sector? | Women/girls make a meaningful contribution to society and the WASH sector. They are recognised as important contributors to programmes and activities | Does the policy recognize that people who menstruate can be productive contributors to society during menstruation? | People who are menstruating make a meaningful contribution to society. |
| Family  resource | Does the policy recognize the value of the family members of women/girls in addressing WASH needs? | The policy recognizes the value of all family members of women/girls, in supporting WASH needs and activities | Does the policy recognize the value of the family members of people who menstruate in addressing MHM needs? | The policy recognizes the value of family members of people who menstruate as a resource for addressing MHM needs. |
| Family  support | Does the policy recognize individual members of women/girls may have an impact on the family members requiring additional support from WASH services? | For example, caring for women/girls with chronic illness may have mental health effects on other family members, such that these family members themselves require support. | Does the policy recognize people who menstruate may have an impact on the family members requiring additional support from MHM services? | For example, caring for menstruating women/girls with chronic illness may have mental health effects on other family members, such that these family members themselves require support. |
| Cultural responsiveness | Does the policy ensure that services respond to the beliefs, values, gender, interpersonal styles, attitudes, cultural, ethnic, or linguistic, aspects of the person, as well as personal safety and dignity? | i) Women/girls are consulted on the acceptability of the service provided ii) Hygiene facilities, goods and services must be respectful of ethical principles and culturally appropriate, i.e. respectful of the culture of women | Does the policy ensure that services respond to the beliefs, values, gender, interpersonal styles, attitudes, cultural, ethnic, or linguistic, aspects of the person, as well as personal safety and dignity? | i) People who menstruate are consulted on the acceptability of the MHM service provided ii) Hygiene facilities, goods and services must be respectful of ethical principles and culturally appropriate, i.e. respectful of the culture of women/girls, LGTBI |
| Accountability | Does the policy specify to whom, and for what, services providers are accountable? | Women/girls have access to internal and independent professional evaluation or procedural safe guard. Do laws/regulations provide mechanisms that ensure complaints are effectively heard? Are there effective complaint mechanisms? Are there judicial bodies that can resolve conflicts? This is evident for both public and private institutions. | Does the policy specify to whom, and for what, MHM services providers are accountable? | People who menstruate have access to internal and independent professional evaluation or procedural safe guard related to MHM. Do laws/regulations provide mechanisms that ensure complaints regarding MHM are effectively heard? Are there effective complaint mechanisms? Are there judicial bodies that can resolve conflicts? This is evident for both public and private institutions. |
| Prevention | Does the policy support women in seeking primary, secondary and tertiary prevention of health conditions associated with WASH? | Includes gendered WASH related illnesses and details on how women/girls can seek primary, secondary and tertiary prevention of associated health conditions, e.g. urinary tract infections | Does the policy support people who menstruate in seeking primary, secondary and tertiary prevention of health conditions associated with menstruation? | Includes complications related to menstruation e.g. reproductive tract infections such as bacterial vaginosis, candidia, and Trichomonas vaginalis (including genital itching, back pain, abdominal pain, pustules over genitalia and abnormal genital discharge) |
| Capacity  building | Does the policy support the capacity building of health workers and of the system that they work in addressing WASH needs of women? | Includes awareness raising among communities and families on the specific WASH issues facing women/girls and potential barriers. | Does the policy support the capacity building of health workers and of the system that they work in addressing MHM needs of people who menstruate? | Includes awareness raising among communities and families on the specific MHM issues facing people who menstruate and potential barriers. |
| Access | Does the policy support women/girls – physical and information access to WASH services? | Women/girls have accessible and safe WASH services within, or in the immediate vicinity, of household, health and educational institution, public institutions and places and workplace. This means that they have separate WASH facilities from men/boys. All information provided on WASH must be understandable and in appropriate format. Does the policy address the needs of both girls and women? | Does the policy support people who menstruate – physical and information access to MHM services? | People who menstruate have accessible and safe MHM facilities within, or in the immediate vicinity, of household, health and educational institution, public institutions and places and workplace. Includes: 1) Private place to change these materials (e.g. toilet), 2. Accessible soap and water for washing the body, 3. Safe and convenient facilities to dispose of used materials. All MHM information must be understandable and in appropriate format. |
| Quality | Does the policy support quality services to women/girls through highlighting the need for evidence-based and professionally skilled practice? Does the policy promote innovation in WASH services for women/girls (e.g. technology)? Does the policy support water that is safe for consumption and other personal uses, so that it presents no threat to human health for women/girls; that sanitation facilities must be hygienically and technically safe to use for women/girls and ensure hygiene, access to water for cleansing and hand washing at critical times | Women/girls are assured of the quality of water supply for consumption and other personal uses; that sanitation facilities are hygienically and technically safe to use by women/girls and that water for hygiene (cleansing, handwashing) is accessible at critical times (i.e. during menstruation, after defecation, changing diapers/nappies, before preparing food and before eating). Services are based on best practice/evidence and support innovative strategies/technology. This includes guidelines | Does the policy support quality MHM services to people who menstruate through highlighting the need for evidence-based and professionally skilled practice? Does the policy promote innovation in MHM services for people? E.g. menstrual product type, information exchange, water for washing the body/menstrual product used; safe and private place to change the menstrual product; methods to dispose of the used menstrual product)? | People who menstruate are assured that disposal facilities are hygienically and technically safe to use and that water for menstrual hygiene is available during menstruation. Services are based on best practice/evidence and support innovative strategies/technology |
| Efficiency | Does the policy support efficiency by providing a structured way of matching WASH system resources with service demands in addressing WASH needs of women/girls? | Are WASH services sustainable for women/girls: Will services still be available in times of financial crisis? Are technology choices appropriate? Do contracts with providers take into account operation and maintenance? Are funds from donors sustainable? | Does the policy support efficiency by providing a structured way of matching MHM system resources with service demands in addressing MHM needs of people who menstruate? | Are MHM services sustainable: Will services still be available in times of financial crisis? Are technology choices and menstrual products appropriate? Do contracts with providers take into account operation and maintenance? Are funds from donors sustainable? |
